# Supplementary material for: Single-cell analysis of gastric signet ring cell carcinoma reveals cytological and immune microenvironment features
Source: Nat Commun. 2023 May 24;14:2985. doi: 10.1038/s41467-023-38426-4 (PMC10209160; doi:10.1038/s41467-023-38426-4)
Supplement: Supplementary file 1 — Supplementary Information [file 41467_2023_38426_MOESM1_ESM.pdf]

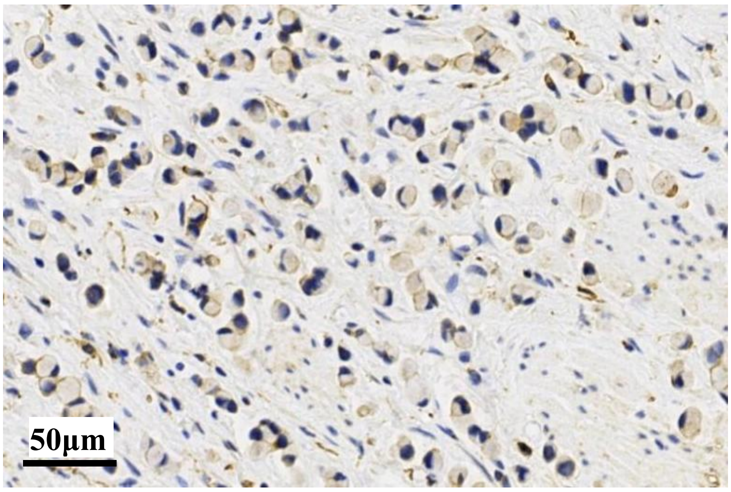


**Figure S1. The positive expression of EpCAM in GSRC.** EpCAM is mainly expressed in the cell membranous and partly in the cytoplasmic (n=30). Scale bar: 50μm.

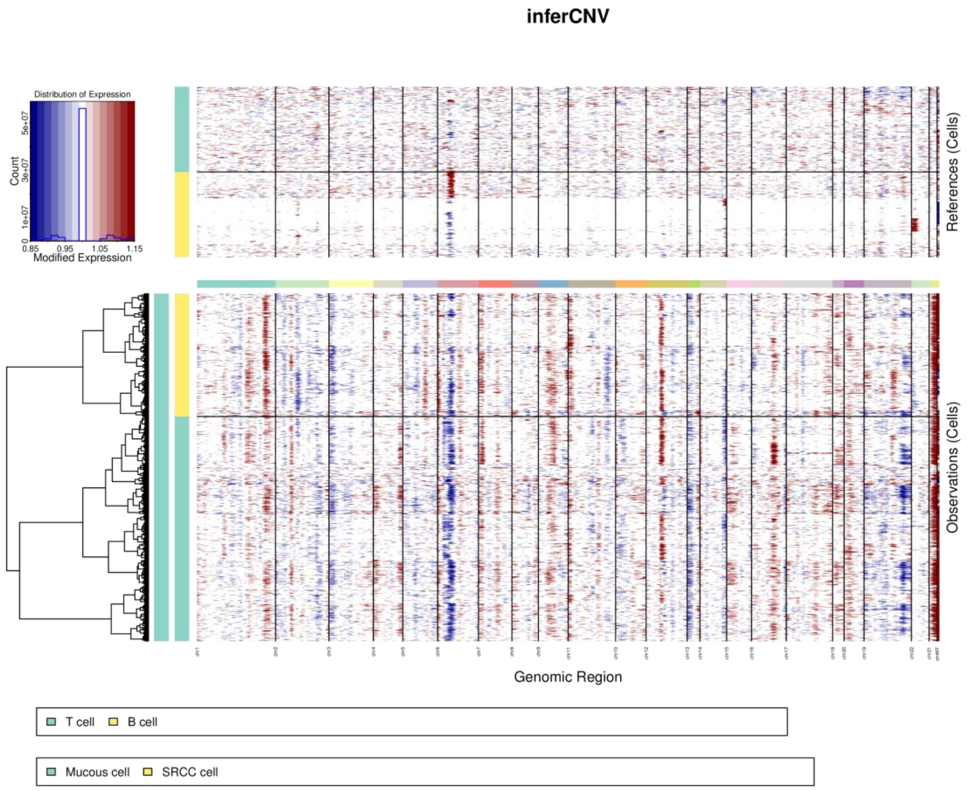


**Figure S2 Heatmap showing large-scale copy number variation for each subclusters of mucous cells and SRCC cells.** Red: amplifications; blue: deletions. Source data are provided as a Source Data file.

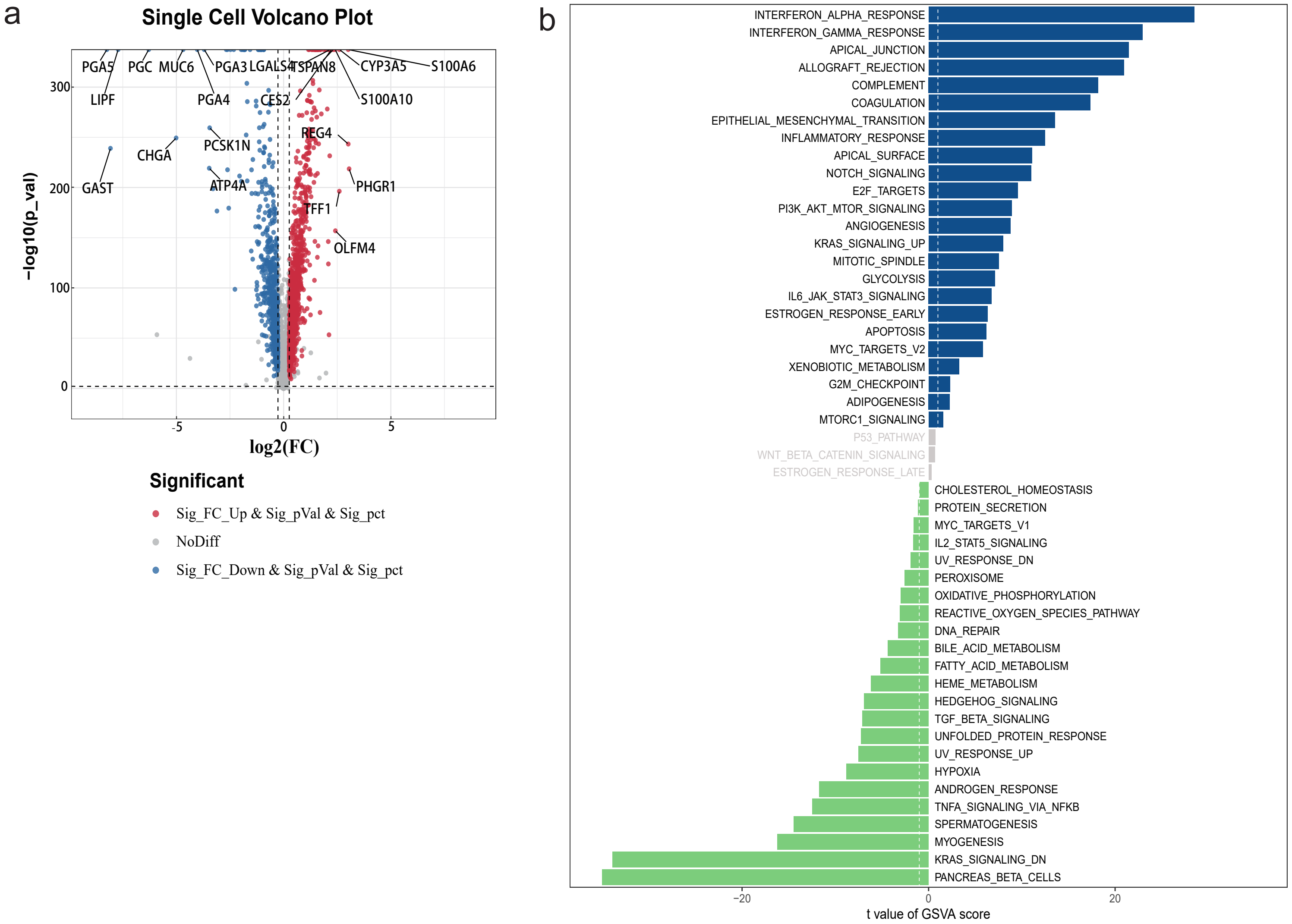


**Figure S3 Differentially expressed genes and GSVA between nonmalignant epithelial cells and M/PDA cells. a** Volcano plot of differentially expressed genes between nonmalignant epithelial cells and M/PDA cells. **b** GSVA of nonmalignant epithelial cells and adenocarcinoma cells. M/DPA cells were mainly enriched in cancer-related signalling pathways, such as epithelial-mesenchymal transition, E2F targets, PI3K/AKT/mTOR signalling, KRAS signalling, etc. GSVA data were plotted according to the t value of limma, and at value > 5 was considered significant. The statistical strategy were two-sided Student's t-test. Source data are provided as a Source Data file.

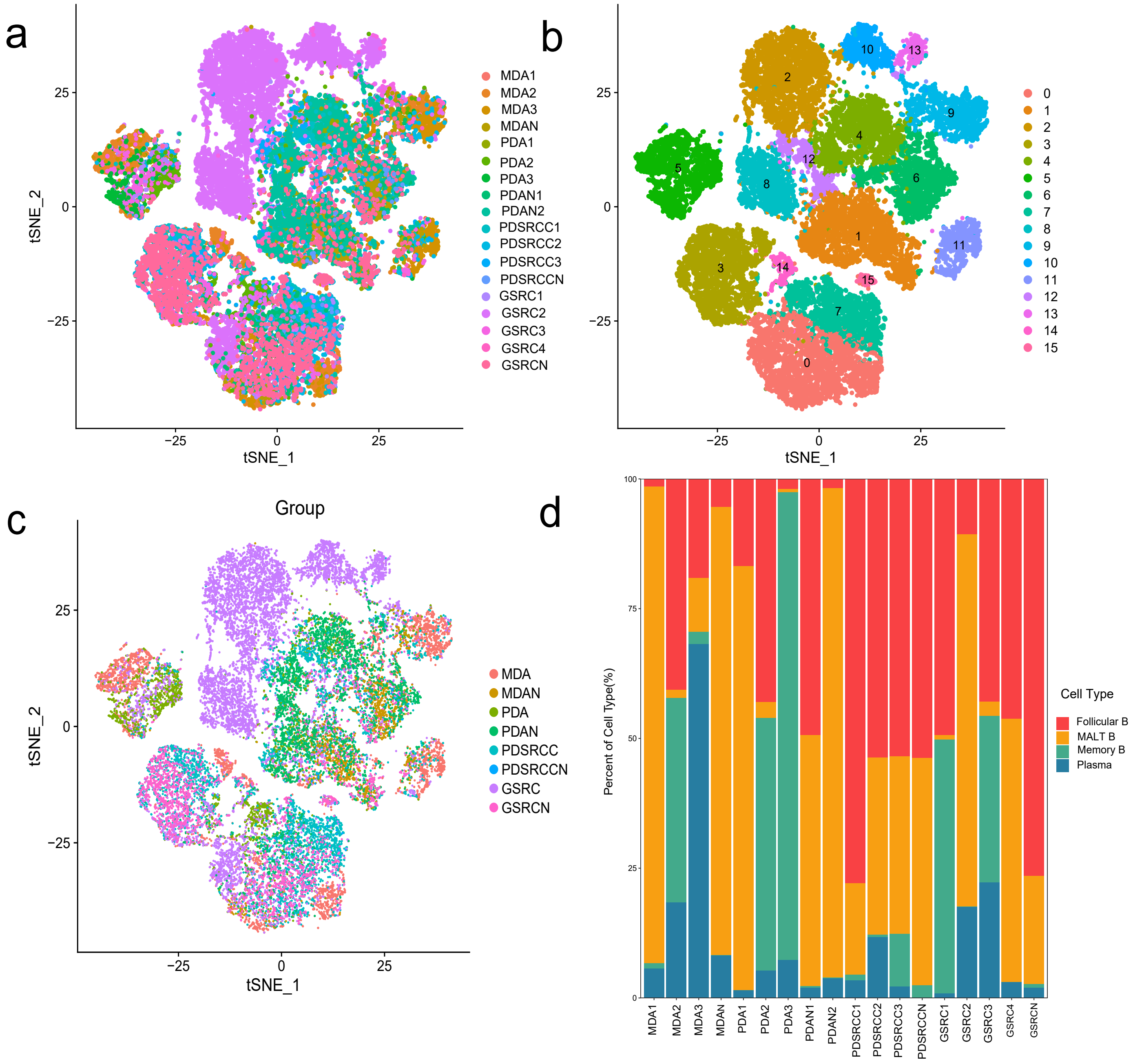


**Figure S4 Classification plots of B cells. a** tSNE plots showing subclusters of B cells (coloured by sample origin). **b** tSNE plots showing subclusters of B cells. **c** tSNE plots showing subclusters of B cells (coloured by grope). **d** Scale plot of subclusters of B cells. Source data are provided as a Source Data file.

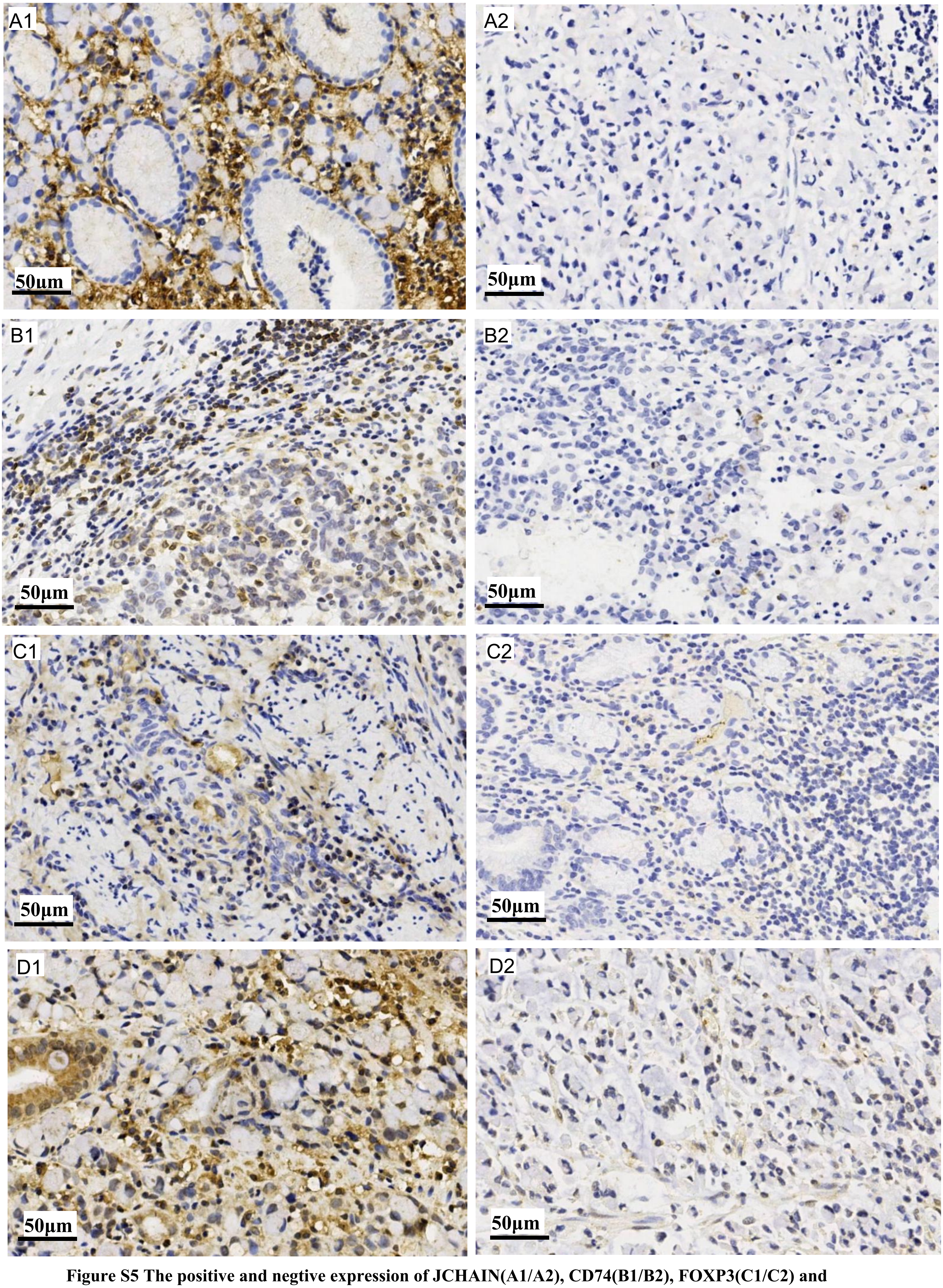


**Figure S5 The positive and negtive expression of JCHAIN(A1/A2), CD74(B1/B2), FOXP3(C1/C2) and KLDR1(D1/D2) in GA (n=30).** Scale bar: 50μm.

**Supplementary Table 1 Table of basic information characteristics of each sample**

| Sample ID | Sex | Age | Tumor or Nomal | Histopathological diagnosis | Lauren's classification | Helicobacter pylori | EBV | MMR | HER-2 | Family History | Cell Number |
|---|---|---|---|---|---|---|---|---|---|---|---|
| MDA1 | Male | 81 | Tumor | MDA | Diffuse | （—） | （—） | dMMR | （—） | （—） | 6015 |
| MDA2 | Male | 68 | Tumor | MDA | Intestinal | （—） | （—） | pMMR | （—） | （—） | 14848 |
| MDA3 | Male | 76 | Tumor | MDA | Intestinal | （—） | （—） | pMMR | （+） | （—） | 13774 |
| PDA1 | Female | 57 | Tumor | PDA | Intestinal | （—） | （—） | pMMR | （—） | （—） | 4684 |
| PDA2 | Male | 52 | Tumor | PDA | Intestinal | （—） | （—） | pMMR | （—） | （—） | 9462 |
| PDA3 | Male | 65 | Tumor | PDA | Diffuse | （—） | （—） | pMMR | （—） | （—） | 3111 |
| PDSRCC1 | Female | 57 | Tumor | PDSRCC | Diffuse | （—） | （—） | pMMR | （—） | （—） | 9546 |
| PDSRCC2 | Female | 61 | Tumor | PDSRCC | Diffuse | （—） | （—） | pMMR | （—） | （—） | 7747 |
| PDSRCC3 | Female | 52 | Tumor | PDSRCC | Diffuse | （—） | （—） | pMMR | （—） | （—） | 6192 |
| GSRC1 | Female | 37 | Tumor | SRCC | Diffuse | （—） | （—） | pMMR | （—） | （—） | 3128 |
| GSRC2 | Female | 48 | Tumor | SRCC | Diffuse | （—） | （—） | pMMR | （—） | （—） | 19955 |
| GSRC3 | Male | 67 | Tumor | SRCC | Mixed | （—） | （—） | pMMR | （—） | （—） | 12830 |
| GSRC4 | Male | 31 | Tumor | SRCC | Diffuse | （—） | （—） | pMMR | （—） | （—） | 6034 |
| MDAN | Male | 81 | Nomal | | | | | | | | 7243 |
| PDAN1 | Female | 57 | Nomal | | | | | | | | 2383 |
| PDAN2 | Male | 52 | Nomal | | | | | | | | 7251 |
| PDSRCCN | Female | 57 | Nomal | | | | | | | | 7914 |
| GSRCN | Female | 37 | Nomal | | | | | | | | 7665 |
