# Supplementary material for: Single-cell analysis of gastric signet ring cell carcinoma reveals cytological and immune microenvironment features
Source: Nat Commun. 2023 May 24;14:2985. doi: 10.1038/s41467-023-38426-4 (PMC10209160; doi:10.1038/s41467-023-38426-4)
Supplement: Supplementary file 3 — Description of Additional Supplementary Files [file 41467_2023_38426_MOESM3_ESM.pdf]

Description of Additional Supplementary Files

File Name: Supplementary Data 1
Description: GSVA of nonmalignant epithelial cells and adenocarcinoma cells
