# Supplementary material for: Single-cell analysis of gastric signet ring cell carcinoma reveals cytological and immune microenvironment features
Source: Nat Commun. 2023 May 24;14:2985. doi: 10.1038/s41467-023-38426-4 (PMC10209160; doi:10.1038/s41467-023-38426-4)
Supplement: Supplementary file 5 — Reporting Summary [file 41467_2023_38426_MOESM5_ESM.pdf]

nature portfolio

Corresponding author(s): Ligang Xing and Jie Chai

Last updated by author(s): Apr 8, 2023

Reporting Summary

Nature Portfolio wishes to improve the reproducibility of the work that we publish. This form provides structure for consistency and transparency in reporting. For further information on Nature Portfolio policies, see our Editorial Policies and the Editorial Policy Checklist.

Statistics

For all statistical analyses, confirm that the following items are present in the figure legend, table legend, main text, or Methods section.

n/a	Confirmed	
☐	☒	The exact sample size (*n*) for each experimental group/condition, given as a discrete number and unit of measurement
☐	☒	A statement on whether measurements were taken from distinct samples or whether the same sample was measured repeatedly
☐	☒	The statistical test(s) used AND whether they are one- or two-sided<br>*Only common tests should be described solely by name; describe more complex techniques in the Methods section.*
☐	☒	A description of all covariates tested
☐	☒	A description of any assumptions or corrections, such as tests of normality and adjustment for multiple comparisons
☐	☒	A full description of the statistical parameters including central tendency (e.g. means) or other basic estimates (e.g. regression coefficient) AND variation (e.g. standard deviation) or associated estimates of uncertainty (e.g. confidence intervals)
☐	☒	For null hypothesis testing, the test statistic (e.g. *F*, *t*, *r*) with confidence intervals, effect sizes, degrees of freedom and *P* value noted<br>*Give P values as exact values whenever suitable.*
☒	☐	For Bayesian analysis, information on the choice of priors and Markov chain Monte Carlo settings
☒	☐	For hierarchical and complex designs, identification of the appropriate level for tests and full reporting of outcomes
☐	☒	Estimates of effect sizes (e.g. Cohen's *d*, Pearson's *r*), indicating how they were calculated

Our web collection on statistics for biologists contains articles on many of the points above.

Software and code

Policy information about availability of computer code

Data collection	Single-cell suspensions were loaded onto a 10X Chromium instrument to capture 8000 single cells according to the manufacturer's instructions for the 10X Genomics Chromium Single-Cell 3' kit (V3). The following cDNA amplification and library construction steps were performed according to the standard protocol. Libraries were sequenced on an Illumina NovaSeq 6000 sequencing system
Data analysis	Single Cell data were analyzed by CellRanger(v7.0.0) to get matrix. And data were analyzed by Seurat (v4.1.1), harmony (v0.1.0) and DoubletFinder (v2.0.3) for further analysis. The plots were performed using the OmicStudio tools created by LC-BIO Co., Ltd (HangZhou, China) at https://www.omicstudio.cn/cell.

For manuscripts utilizing custom algorithms or software that are central to the research but not yet described in published literature, software must be made available to editors and reviewers. We strongly encourage code deposition in a community repository (e.g. GitHub). See the Nature Portfolio guidelines for submitting code & software for further information.

Data

Policy information about availability of data

All manuscripts must include a data availability statement. This statement should provide the following information, where applicable:

- Accession codes, unique identifiers, or web links for publicly available datasets
- A description of any restrictions on data availability
- For clinical datasets or third party data, please ensure that the statement adheres to our policy

The raw scRNA-seq data used in this study are available in the Genome Sequence Archive (Genomics, Proteomics & Bioinformatics 2021) in the National Genomics Data Center (Nucleic Acids Res 2022), China National Center for Bioinformation/Beijing Institute of Genomics, Chinese Academy of Sciences database under accession code GSA-Human: HRA003647 (https://ngdc.cncb.ac.cn/gsa-human). We have all relevant approvals from China's Ministry of Science and Technology (Filing number: 2023BAT0334). The raw RNA-seq data generated in this study have been deposited in the Genome Sequence Archive (Genomics, Proteomics & Bioinformatics 2021) in the National Genomics Data Center (Nucleic Acids Res 2022), China National Center for Bioinformation/Beijing Institute of Genomics, Chinese Academy of Sciences database under accession code GSA-Human: HRA003650 [https://ngdc.cncb.ac.cn/gsa-human]. We have all relevant approvals from China's Ministry of Science and Technology (Filing number: 2023BAT0333). The published data used for validation of the expression of MSMB in cell lines in this manuscript were retrieved from DepMap databases (https://depmap.org/portal/Interactive). The data are provided in the form of a single Excel file and placed in multiple label files in a compressed folder. This file or folder was also named “Source Data”. Source data are provided in this manuscript. The remaining data are also available in this manuscript.

Human research participants

Policy information about studies involving human research participants and Sex and Gender in Research.

Reporting on sex and gender	This study has not involved humans categorized and no sex- and gender-based analyses have been performedIt �did not involve special research on human populations (including reporting standards).
Population characteristics	The gastroscopic biopsy pathology of the enrolled patients at the first diagnosis was GA, and they had not received anti-tumor treatments such as radiotherapy, chemotherapy and immunotherapy before surgery, and did not have tumors inother parts. All the patients in this study are Han people. 13 patients were prospectively enrolled for scRNA-seq, including 7 males and 6 females with median age of 63 and 51 years old(their grouping was mainly based on the degree of differentiation and the content of signet ring cells, and no gender-based analysis was performed).
Recruitment	All donors are recruited and managed by Shandong Cancer Institute and Jinan Central Hospital. Thirteen patients who were pathologically diagnosed with advanced gastric signet ring cell content or gastric adenocarcinoma were enrolled in this study.
Ethics oversight	The study protocol was approved by the ethics committee of Shandong Cancer Institute and Jinan People’s Hospital for Human Study and conducted according to the principles of the Declaration of Helsinki.

Note that full information on the approval of the study protocol must also be provided in the manuscript.

Field-specific reporting

Please select the one below that is the best fit for your research. If you are not sure, read the appropriate sections before making your selection.

☒ Life sciences ☐ Behavioural & social sciences ☐ Ecological, evolutionary & environmental sciences

For a reference copy of the document with all sections, see nature.com/documents/nr-reporting-summary-flat.pdf

Life sciences study design

All studies must disclose on these points even when the disclosure is negative.

Sample size	A total of 13 gastric cancer patients for 10x Genomics scRNA-seq analysis were involved in this study. The patients were grouped mainly based on the degree of differentiation and signet ring cell content, and no gender-based analysis was performed. In the validation cohort, 60 patients were retrospectively enrolled for immunohistochemistry. All patients received surgical resection.
Data exclusions	All criteria for data exclusion were pre-established. We eliminated low quality cells and checked with feature gene expressions to remove unqualified events including doublets, contaminating cells and cells with high fraction expression of dissociation genes.No tumor samples were excluded.
Replication	The study includes multiregional samples of a tumor lesion from patients. Each region sampled from the same lesion can be interpreted as a biological replicate of the tumor analyzed. All attempts at replication were successful.
Randomization	All patients involved in this study were primary gastric cancer patients and received surgical resection. And the divided groups were determined according to clinical diagnosis.

Blinding

Blinding was not relevant, since there were no expected outcomes or pre-defined outcome measures.

Reporting for specific materials, systems and methods

We require information from authors about some types of materials, experimental systems and methods used in many studies. Here, indicate whether each material, system or method listed is relevant to your study. If you are not sure if a list item applies to your research, read the appropriate section before selecting a response.

Materials & experimental systems

n/a	Involved in the study
☐	☒ Antibodies
☐	☒ Eukaryotic cell lines
☒	☐ Palaeontology and archaeology
☒	☐ Animals and other organisms
☒	☐ Clinical data
☒	☐ Dual use research of concern

Methods

n/a	Involved in the study
☒	☐ ChIP-seq
☒	☐ Flow cytometry
☒	☐ MRI-based neuroimaging

Antibodies

Antibodies used

anti-EpCAM (rabbit, 1:400, bs-0593R, Bioss, China), anti-TTF1 (rabbit, 1:1000, 66034-1-IG, 2C8F3, PROTEINTECH GROUP, USA), anti-TTF2 (rabbit, 1:100, 13722-1-AP, PROTEINTECH GROUP, USA), anti-MUC5AC (mouse,1:200, GB14112, l107, Servicebio, China), anti-MUC6 (mouse, 1:50, GB14113, l108, Servicebio, China), anti-JCHAIN (rabbit, 1:1000, bsm-60277R, J2H8, Bioss, China), anti-MSMB (rabbit, 1:200, bs-19185R, Bioss, China), anti-CD74 (mouse, 1:1000, GB121179, 3H10A4, Servicebio, China), anti-FOXP3 (rabbit, 1:400, GB11093, Servicebio, China), antiKLRD1 (rabbit, 1:100, DF6773, Affinity Biosciences, USA).

Validation

1.EpCAM: ①.Product application: ELISA=1:5000-10000 IHC-P=1:100-500IHC-F=1:100-500 IF=1:100-500; not yet tested in other applications; optimal dilutions/concentrations should be determined by the end user. ②.Database application verification: [IF=4.35] Xiuxiu Wang. et al. Evaluation of the Effects of Different Dietary Patterns on Breast Cancer: Monitoring Circulating Tumor Cells. Foods. 2021 Sep;10(9):2223 IF; mouse. [IF=9.518] Fan Y et al. High-sensitive and multiplex biosensing assay of NSCLC-derived exosomes via different recognition sites based on SPRi array. Biosens Bioelectron. 2020 Apr 15; 154:112066. WB&SPRi biosensor; Human.
2.TTF1: ①.Product application: WB=1:500-2000 IHC-P=1:100-500 IHC-F=1:100-500 ICC=1:100-500 IF=1:100-500 not yet tested in other applications. ②.Database application verification: [IF=4.872] Dong X et al. PM2.5 disrupts thyroid hormone homeostasis through activation of the hypothalamic-pituitary-thyroid (HT) axis and induction of hepatic transthyretin in female rats 2.5Ecotoxicol Environ Saf.2021 Jan 15;208:111720. IHCAWB; Rat. [IF=3.14] Huang, Huibin, et al. "Upregulation of thyroid transcription factor-1 and human leukocyte antigen class I in Hashimoto's disease providing clinical evidence for possible triggering autoimmune reaction." European Journal of Endocrinology 164.5 (2011): 795-800. WB, IHC-P; Human. [IF=1.38] Vadasz, Stephanie, et al. "Second and third trimester amniotic fluid mesenchymal stem cells can repopulate a decellularized lung scaffold and express lung markers." Journal of Pediatric Surgery (2014). Human.
3.TTF2: ①.Product application: WB=1:500-2000 ELISA=1:5000-10000 IHC-F=1:100-500 not yet tested in other applications; optimal dilutions/concentrations should be determined by the end user. ②.Database application verification: [IF=3.644] Ran Qet al. Generation of Thyroid Tissues from Embryonic Stem Cells via Blastocyst Complementation In Vivo Front Endocrinol (Lausanne).2020 Dec 14;11:609697. F Mouse. [IF=3.58] Wang, Qi, et al. "Methamphetamine induces hepatotoxicity via inhibiting cell division, arresting cell cycle and activating apoptosis: In vivo and in vitro studies." Food and Chemical Toxicology (2017). WB; Rat.
4.MUC5AC: ①.Product application: ELISA-1:5000-10000IHC-P=1:100-500 IHC-F=1:100-500IF=1:100-500 not yet tested in other applications; optimal dilutions/concentrations should be determined by the end user. ②.Database application verification: [IF=7.076] Annika Sünderhauf. et al. Loss of Mucosal p32/C1qR/HABP1 Triggers Energy Deficiency and Impairs Goblet Cell Differentiation in Ulcerative Colitis. Cell Mol Gastroenter. 2021 Jan; ELISA, IHC; Mouse. [IF=8.46] Taki, K., et al. "GNASR201H and KrasG12D cooperate to promote murine pancreatic tumorigenesis recapitulating human intraductal papillary mucinous neoplasm." Oncogene 35.18 (2016): 2407-2412. IHC-P; Mouse.
5. MUC6: ①.Product application: IHC-P=1:100-500 IHC-F=1:100-500 ICC=1:100-500 IF=1:100-500 not yet tested in other applications; optimal dilutions/concentrations should be determined by the end user. ②.Database application verification: no
6?JCHAIN: ①. Product application: WB=1:500-2000 ELISA=1:5000-10000 IHC-P=1:100-500 IHC-F=1:100-500 IF=1:50-200 not yet tested in other applications; optimal dilutions/concentrations should be determined by the end user. ②.Database application verification: no
7.MSMB: ①.Product application: ELISA=1:5000-10000 IHC-P=1: 100-500 IHC-F=1:100-500
CC=1:100-500 IF=1:100-500 not yet tested in other applications. optimal dilutions/concentrations should be determined by the end user. ②.Database application verification: no
8.CD74: ①.Product application: ELISA=1:5000-10000 IHC-P=1:400-800 not yet tested in other applications; optimal dilutions/concentrations should be determined by the end user. ②. Database application verification: no
9.FoxP3 ①.Product application: ELISA=1:5000-10000 IHC-P=1:100-500 IHC-F-1:100-500 Flow-Cyt=µ3ug/Test IF=1:100-500 not yet tested in other applications; optimal dilutions/concentrations should be determined by the end user. ②.Database application al. verification: [IF=7.367] Yitian Du. et al. Engineered Microglia Potentiate the Action of Drugs against Glioma Through Extracellular Vesicles and Tunneling Nanotubes. 2021 Feb 28 IF, IHC; Mouse. [IF =3.208] Matsubara et al. Immune activation during the implantation phase causes preeclampsia-like symptoms via the CD40-CD40 ligand pathway in pregnant mice. (2016) Hypertens.Re.39:407-14 IHC; Mouse.
10.KLRD1: ①.Product application: This reagent has been tested for flow cytometric analysis. It is recommended that this reagent

should be titrated in each testing system to obtain optimal results. WB : 1:500-1:1000. ②.Database application verification: K-562 cells were subjected to SDS PAGE followed by western blot with 13332-1-AP (CD94 antibody at dilution of 1:600 incubated at room temperature for 1.5 hours.

Eukaryotic cell lines

Policy information about cell lines and Sex and Gender in Research

Cell line source(s)	MKN-45 (CBP60488)and NUGC-4(CBP60493) cells were purchased from Nanjing Kebai Biotechnology Co., Ltd (Nanjing, China). MKN-45 cells : Species: human, Gender: 62 year-old female. NUGC-4 cells :Species: human, Gender: 35year-old female.
Authentication	MKN-45 and NUGC-4 cells were authenticated using short tandem repeat analysis.
Mycoplasma contamination	MKN-45 and NUGC-4 cells have no mycoplasma contamination.
Commonly misidentified lines (See ICLAC register)	No commonly misidentified cell lines were used.
